# Ethoxy Groups on ZrO_2_, CuO, and CuO/ZrO_2_ Studied by IR Spectroscopy

**DOI:** 10.3390/molecules27154790

**Published:** 2022-07-26

**Authors:** Jerzy Podobiński, Michał Śliwa, Jerzy Datka

**Affiliations:** Jerzy Haber Institute of Catalysis and Surface Chemistry, Polish Academy of Sciences, Niezapominajek 8, 30-239 Krakow, Poland; jerzy.podobinski@ikifp.edu.pl

**Keywords:** IR spectroscopy, ethoxy groups, ZrO_2_, CuO, CuO/ZrO_2_

## Abstract

The formation, properties, decomposition and reactions of ethoxy groups on ZrO_2_, CuO, and CuO/ZrO_2_ were followed by IR spectroscopy. The reaction of ethanol with terminal Zr-OH groups leads to the formation of monodendate ethoxy groups (type I), whereas the reaction of ethanol with tribridged Zr-OH grups results in the formation of bidendate ethoxyls (type II). In both cases, water is produced. Ethoxy groups of type II were also formed on CuO. The type of the surface species detected after interaction of ethanol with CuO/ZrO_2_ was the same as detected for both oxides (i.e., ZrO_2_ and CuO) separately. This suggests that no new phase was formed in the mixed oxide system. At higher temperatures, ethoxy groups were oxidized forming acetate ions. Gaseous ethanol present in the cell was oxidized to acetaldehyde without the intermediacy of ethoxy groups.

## 1. Introduction

Ethanol has drawn considerable attention as a substrate for hydrogen production. The main advantages of ethanol lie in the facts that it is non-toxic and can be easily handled, stored and transported. Moreover, ethanol can be easily produced from renewable natural resources (biomass). Hydrogen may be produced from ethanol by steam reforming [1], oxidative steam reforming [2], partial oxidation [3], and dehydrogenation/decomposition [4] processes. The dehydrogenation and decomposition of ethanol are specially interesting because not only clean hydrogen but also other chemicals that are valuable for industry such as acetaldehyde, acetic acid, ethyl acetate, diethyl ether, and carbon monoxide can be produced.

Generally, ethanol undergoes two main processes on various catalysts, which are oxidation and dehydrogenation to acetaldehyde as well as dehydration to ethylene. The distribution of these two products depends on the ratio of acidic to basic sites. Acid sites catalyze ethanol dehydration to ethene whereas basic sites promote the dehydrogenation to acetaldehyde. The dehydration occurs on solid acids (zeolites [5], alumina [6,7], SAPO [8] or heteropolyacids [9]. On the other hand, acetaldehyde or ethyl acetate are obtained through supported transition metals (Cu, Ni, Co, Fe) [10,11,12], and noble metals (Pt, Pd, Ru) catalysts [13].

According to literature data, Cu- and Ni-based catalysts exhibit good activity in the ethanol conversion but they differ in the final main products. This is due to the fact that Cu promotes C–H cleavage, leading to acetaldehyde, whereas Ni facilitates C–C bond dissociation resulting in CO and CH_4_ [14]. The formation of ethoxy species is found during dehydrogenation/decomposition of ethanol over catalysts containing transition metals. These species can be converted further to different products depending on the nature of used catalyst [13,15,16,17].

Alkoxy (methoxy and ethoxy) groups have been the subject of numerous studies [18,19,20,21,22,23,24,25,26,27,28]. Based on the literature, one can say that there are several mechanisms, which explain the formation of these groups. The mechanism of condensation, which is accompanied by release of water molecules, was proposed: M-OH + HO-R = M-O-R + H_2_O (where R is CH_3_ or C_2_H_5_ group) [20,28] in the case of oxides, whose surface is enriched in the hydroxyl groups.

Another mechanism [18,20,23,24,27] assumes the reaction of M-O-M entities with alcohol leading to the formation of alkoxy groups and surface hydroxyl M-O-M + HO-R = M-O-R + M-OH. Moreover, Bianchi et al. [20] proposed a mechanism, according to which the bridge of M-O-M reacted with two alcohol molecules: M-O-M + 2 R-OH = 2 M-O-R + H_2_O. The formed alkoxy groups can undergo further reactions, giving acetaldehyde (for ethoxy groups), carboxy and carbonate species, as well as CO, CO_2_, CH_4_ and hydrogen [19,20,21,22,23,24,25,26,27,28].

Since ZrO_2_ and CeO_2_ are components of numerous catalysts in the processes of ethanol conversion to hydrogen, most of the studies of formation and reactivity of ethoxy groups were done with these oxides [18,19,20,21,22,24,25,26,27,28].

In our previous research [29] we followed the transformations of ethanol over CuO, CuO/ZrO_2_, CuO/ZrO_2_/ZnO, CuO/ZrO_2_/NiO and CuO/ZrO_2_/ZnO/NiO. For IR studies, these catalysts were diluted in SiO_2_. It has been found that for all these catalysts ethanol was transformed to acetaldehyde, but the mechanism of this process was different for various catalysts. In the case of CuO, acetaldehyde was obtained exclusively by oxidation. For all other catalysts both oxidation and dehydrogenation took place (hydrogen was produced). For all catalysts, except for CuO, acetaldehyde was further oxidized to acetic acid, which was subsequently transformed to acetone.

In this study we followed, by IR spectroscopy, the formation, properties and reactivity of ethoxy groups formed by the reaction of ethanol with ZrO_2_, CuO, and CuO/ZrO_2_. We paid special attention to the role of ethoxy groups in the formation of acetaldehyde and acetate species.

## 2. Results and Discussion

### 2.1. X-ray Diffraction Analysis

**Figure 1:**
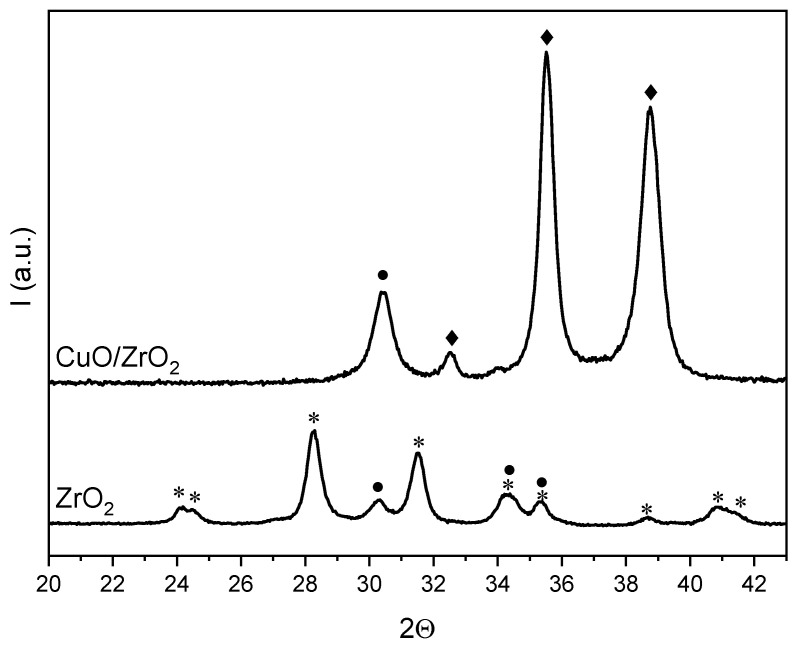
XRD patterns for ZrO_2_ and CuO/ZrO_2_: m-ZrO_2_ (*), t-ZrO_2_ (●), CuO (♦).

The XRD diffractograms of ZrO_2_ and CuO/ZrO_2_ are presented in Figure 1. In the case of pure ZrO_2_, two crystalline structures are visible, i.e., the monoclinic phase (m-ZrO_2_, main signals at 2θ = 28.2° and 2θ = 31.5) and tetragonal phase (t-ZrO_2_, main signal at 2θ = 30.2°), whose proportions correspond to 92 and 8% by volume fraction, respectively. Since the ZrO_2_ is the mixture of two phases, the crystallite sizes of m-ZrO_2_ and t-ZrO_2_ were calculated separately according to the appropriate diffraction peaks, which are at 2θ = 28.2° and 2θ = 30.2°, respectively. The obtained values of crystallite size for m-ZrO_2_ is 18.9 nm, whereas for t-ZrO_2_ it is 15.6 nm.

For the CuO/ZrO_2_ catalyst, two separated phases are present, which are CuO (main signals at 2θ = 35.5° and 38.8°, tenorite structure) and t-ZrO_2_ (main signal at 2θ = 30.2°). The signal assigned to t-ZrO_2_ is of higher intensity in comparison with pure ZrO_2_. This is due to the higher concentration of the t-ZrO_2_ phase in CuO/ZrO_2_. The calculation of crystallite sizes for CuO and t-ZrO_2_ were made based on the signals at 2θ = 35.5° and 2θ = 30.2, respectively. The obtained values of crystallite size for CuO is 19.4 nm and for t-ZrO_2_ it is 13.6 nm.

### 2.2. Reaction of Ethanol with ZrO_2_—Formation of an Ethoxy Group

**Scheme 1:**
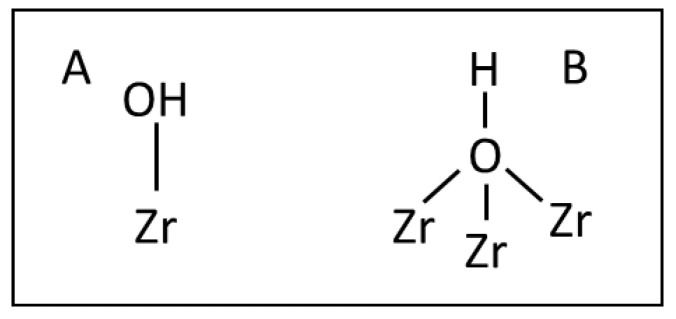
Monobridged (**A**) and tribridged (**B**) hydroxyl species.

**Figure 2:**
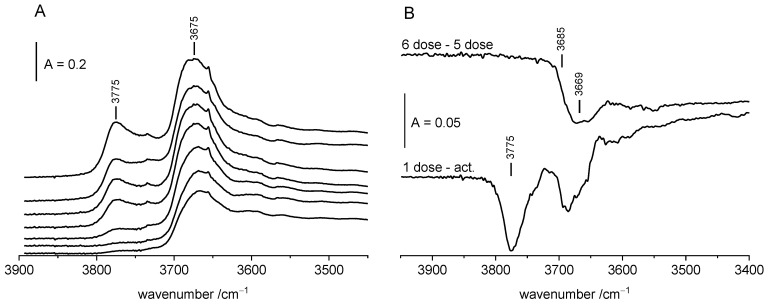

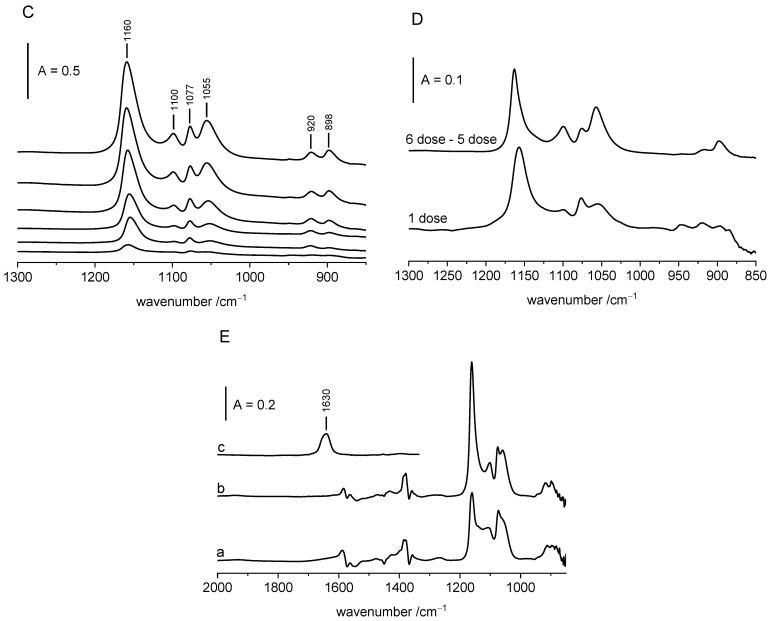
(**A**)—The spectra of OH groups on ZrO_2_ upon adsorption of ethanol (from bottom to top increasing loading of ethanol). Each spectrum was recorded upon the adsorption of one dose of ethanol (ca. 5 µmol of ethanol/100 mg of ZrO_2_. (**B**)—OH groups on ZrO_2_, difference spectra: activated ZrO_2_ minus 1st dose of ethanol and 6th dose minus 5th dose. (**C**)—alkoxy groups on ZrO_2_ (from bottom to top increasing loading of ethanol). Each spectrum was recorded upon the adsorption of one dose of ethanol (ca. 5 µmol of ethanol/100 mg of ZrO_2_. (**D**)—alkoxy groups on ZrO_2_, 1st dose of ethanol, 6th dose minus 5th. (**E**)—alkoxy groups on ZrO_2_ (a), upon desorption at 370 K (b), spectrum of water desorbed from ZrO_2_ and readsorbed on zeolite NaY. All the spectra were recorded at RT.

The IR spectrum (Figure 2A) of ZrO_2_ dehydrated at 470 K shows two distinct OH bands at 3675 and 3775 cm^−1^ as well as a weak band at 3733 cm^−1^. According to several authors [30,31,32,33,34,35] these bands were assigned to terminal (3775 cm^−1^), dibridged (3733 cm^−1^) and tribridged (3675 cm^−1^) hydroxyls, bonded to, respectively, one, two or three Zr atoms (Scheme 1).

According to [33] terminal hydroxyls are single cations at oxygen lattice faces, whereas the multicoordinated hydroxyls are located at low index faces. Several authors [30,35,36] revealed that two types of tribridged hydroxyls slightly differ in their acidity.

The reaction of ethanol with ZrO_2_ (Figure 2A) resulted in the vanishing of 3775 cm^−1^ OH band and in the decrease of the 3675 cm^−1^ one. The experiments of the adsorption of consecutive doses of ethanol (Figure 2A) followed by heating to 420 K and evacuation at 470 K evidenced that 3775 cm^−1^ terminal hydroxyls were more reactive than 3675 cm^−1^ ones (tribridged), i.e., the 3775 cm^−1^ band decreased in the first order. These results agree with the observation of Bensitel et al. [19], who did also reveal that terminal Zr-OH groups reacted with ethanol in the first order. The same authors reported also that only terminal Zr-OH groups reacted also with CO_2_-forming hydrogencarbonate species.

Having compared the different spectra presented in Figure 2B, it evidences that the band of tribridged hydroxyls (3675 cm^−1^) is complex. It is composed of two submaxima at 3669 and 3685 cm^−1^. High frequency hydroxyls (3685 cm^−1^) react with ethanol at room temperature whereas low frequency ones (3669 cm^−1^) are less reactive.

The spectra of ethoxy groups were considered in the region 800–1300 cm^−1^. (below 800 cm^−1^ the spectrum was illegible). Reaction of ethanol with ZrO_2_ produces ethoxy groups for which there were six bands: 898, 920, 1055, 1077, 1100 and 1160 cm^−1^ were characteristic. The interpretation of these bands was discussed by Bonsitel et al. [19] as well as Silchenkova et al. [28]. Bonsitel et al. [19] proposed that 920, 1075 and 1160 cm^−1^ bands were due to monodendate ethoxy groups (type I) formed by the reaction of ethanol with 3775 cm^−1^ hydroxyls, and the 895, 1055 and 1100 cm^−1^ bands were due to bidendate ethoxy groups (type II) formed by the reaction of ethanol with 3670 cm^−1^ hydroxyls. Another interpretation was proposed by Silchenkova et al. [28] who assigned 1100 and 1150 cm^−1^ bands to the same species, i.e., linear (monodendate) ethoxy groups, and 1060 cm^−1^ to another species, i.e., bridged (bidendate) ethoxy groups.

**Scheme 2:**
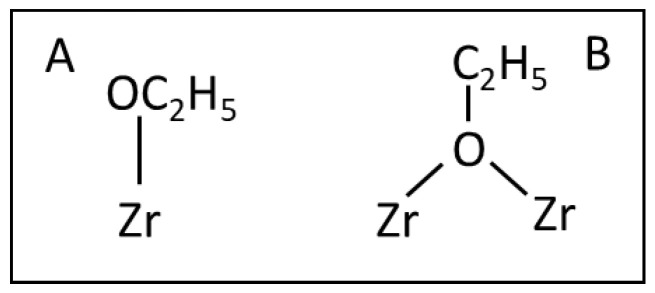
Monodendate (**A**) and bidendate (**B**) ethoxyl species.

The results obtained in our study confirm the interpretation of Bonsitel et al. [19]. According the data presented in Figure 2D, the 920, 1077 and 1160 cm^−1^ bands (type I) grow “in concert” at low coverage, whereas at higher coverage, the 898, 1055 and 1100 cm^−1^ ones (type II) grow also “in concert”; therefore each of these “trios” represent one kind of ethoxy groups. In another words, the 1100 and 1160 cm^−1^ bands are due to two different kinds of ethoxy groups. The analysis of the difference spectra presented in Figure 2B,D suggests that type I ethoxyls (monodendate) were produced by the reaction of ethanol with terminal hydroxyls and type II (bidendate) alkoxyls were produced with tribridged hydroxyls (Scheme 2). The same interpretation was proposed by Bonsitel et al. [19].

As mentioned in the Introduction section, several mechanisms of the formation of alkoxy (methoxy or ethoxy) groups on oxide surfaces were considered. Most of the authors [19,20,23,24,27] assumed the reaction of hydrogen from alcohol with surface oxygen with the formation of surface hydroxyls: M-O-M + HO-R = M-O-R + M-OH. Another mechanism [20,28]: M-OH + HO-R = M-O-R + H_2_O assumes the consumption of surface hydroxyls and the formation of water. The results obtained in this study confirm the latter mechanism. According to the data presented in Figure 2, Zr-OH is consumed and not formed (as assumed in former mechanism). Another argument supporting later mechanism is the formation of water. The formation of water was visible in the experiment, in which the products of reaction of ethanol over ZrO_2_ were desorbed in liquid nitrogen trap and next adsorbed on freshly dehydrated zeolite NaY. The spectrum of zeolite with the reaction products (Figure 2E) shows the 1630 cm^−1^ band of the deformation vibration of water, evidencing that ethanol reacts with surface hydroxyls forming ethoxy groups and water. The IR bands of water above 3000 cm^−1^ superpose the band of hydroxyls in ethanol, so the spectrum in this region was not considered.

### 2.3. Decomposition of Ethoxy Groups on ZrO_2_

**Figure 3:**
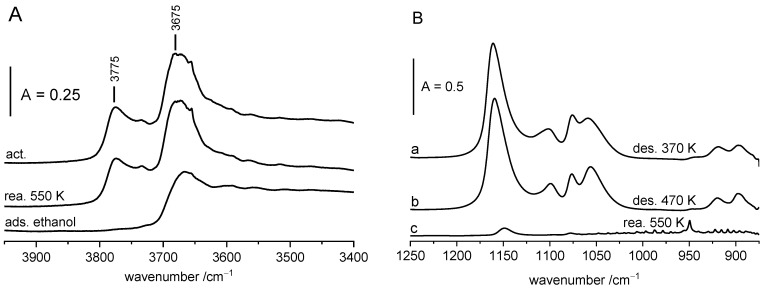
Decomposition of ethoxy groups on ZrO_2_. (**A**)—OH groups on ZrO_2_ activated upon the adsorption of ethanol (ca. 30 µmol of ethanol/100 mg ZrO_2_) and upon the reaction of adsorbed ethanol at 550 K. (**B**)—ethoxy groups on ZrO_2_ upon desorption at 370 K (a) and at 470 K (b), as well as upon reaction at 550 K (c). All the spectra were recorded at RT.

Ethoxy groups on ZrO_2_ are relatively stable; the intensity of their bands practically does not change upon the evacuation at 470 K (Figure 3). They decompose above this temperature, producing ethene (oscillation–rotational bands in the 900–1050 cm^−1^ region Figure 3B, spectrum c). The spectrum is identical with the spectrum of pure ethane. All the hydroxyl groups are restored (Figure 3A).

### 2.4. Interaction of Ethanol with CuO

**Figure 4:**
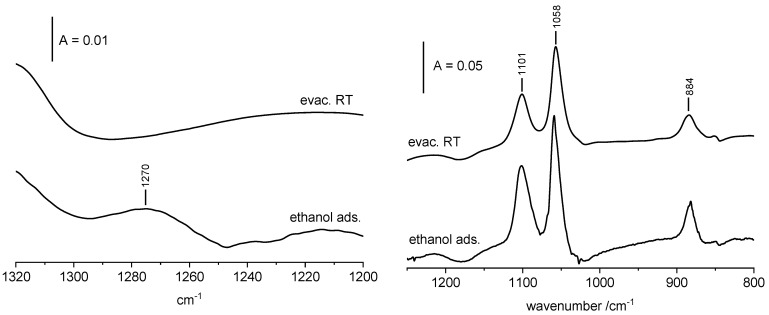
The spectra recorded upon the adsorption of ethanol (ca. 15 Tr. in gas phase) on CuO and evacuation at RT. The spectrum of gaseous ethanol was subtracted.

**Figure 5:**
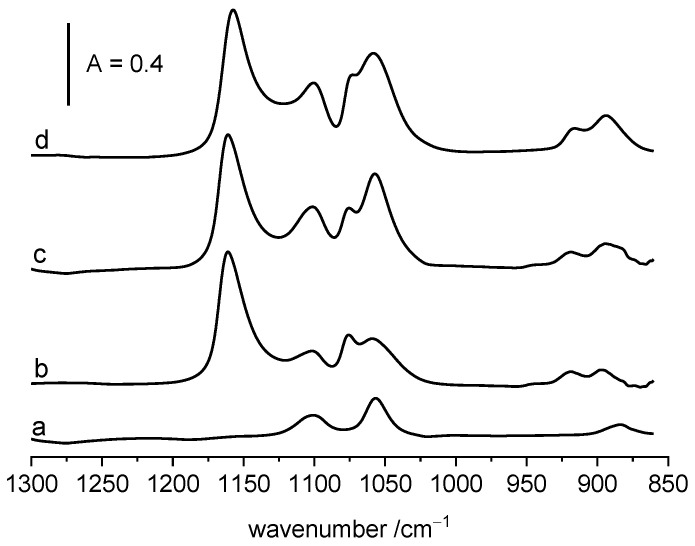
The spectra of the species formed by ethanol adsorption on CuO (a), ZrO_2_ (b) and CuO/ZrO_2_ (c) as well as the result of the addition of spectra a and b.

The spectra recorded upon the adsorption of ethanol on CuO at room temperature followed by evacuation at RT are presented in Figure 4 and Figure 5. Adsorption of ethanol on CuO results in appearing of three relatively weak bands at 884, 1058 and 1101 cm^−1^. The assignment of these bands was the subject of our consideration. Two possibilities were considered. One of them assumes that they are due to ethoxy groups of type II, and another one assigns them to adsorbed molecular ethanol. The analysis of band positions does not give definitive answers, because the positions of the bands of both ethoxy groups of type II on ZrO_2_ and of liquid ethanol are very close (898, 1055 and 1100 cm^−1^ for ethoxy groups on ZrO_2_ and 882, 1050 and 1090 cm^−1^ for ethanol). In order to get more information, we analyzed carefully the region between 1200–1300 cm^−1^ in which the very weak band at ca. 1270 cm^−1^ of the bending vibration of the OH groups in molecular ethanol [37] appears. The appearance of the 1270 cm^−1^ is evidence of the presence of molecular ethanol. The spectrum presented in Figure 4 was recorded upon the adsorption of excess of ethanol (ca. 5 Tr in gas phase) and the subsequent subtraction of the spectrum of gas phase. The band at 1270 cm^−1^ is present (together with the bands 884, 1058 and 1101 cm^−1^) indicating the presence of adsorbed ethanol. The evacuation at room temperature causes the disappearing of the 1270 cm^−1^ band (Figure 4, top spectrum) evidencing that molecular ethanol was desorbed. The bands at 884, 1058 and 1101 cm^−1^ decreased only a little due to the desorption of ethanol, but they are still present at the absence of molecular ethanol, evidencing that they can be assigned to ethoxy groups of type II (bidendate) on CuO.

In order to know what the mechanism of ethoxyl groups formation on CuO is, we wanted to know if water was produced by this reaction. Therefore, we realized an experiment in which the products of reaction of ethanol on CuO were adsorbed on dehydrated zeolite (similarly as it was done for ZrO_2_), but in this case (alike for ZrO_2_) no water was detected. Therefore, it may be supposed that ethoxy groups are not formed by the reaction of ethanol with surface Cu-OH (as is was found for ZrO_2_) but by the reaction of ethanol with Cu-O-Cu entities according to the hypothetical scheme: C_2_H_5_-OH + Cu-O-Cu = C_2_H_5_O-Cu + Cu-OH. Unfortunately, the formation of Cu-OH cannot be established because of very poor transmittance of CuO wafer in the OH region.

### 2.5. Ethoxy Groups on CuO/ZrO_2_

The spectrum recorded upon the adsorption of ethanol on CuO/ZrO_2_ at room temperature followed by evacuation at 370 K is presented in Figure 5. The spectra of ethanol adsorbed at the same conditions on pure ZrO_2_ and on CuO are presented as well. Line c in Figure 5 is the result of addition of the spectra of ethanol adsorbed on both ZrO_2_ and on CuO. The appearance of this line is similar to the spectrum of ethoxy groups on CuO/ZrO_2_. This suggests that on CuO/ZrO_2_ we have the same species as on each of the individual oxides and no new crystalline phase is formed. This conclusion agrees with the results of our earlier XRD studies, which showed that only CuO and t-ZrO_2_ phases are present in CuO/ZrO_2_ catalyst [38]. Similarly, as for pure ZrO_2_, the reaction of ethanol with CuO/ZrO_2_ produces water. It was evidenced in an experiment similar as for ZrO_2_ in which the products of reaction were sorbed in zeolite NaY (spectrum not shown).

### 2.6. Reaction of Alkoxy Groups on ZrO_2_, CuO and CuO/ZrO_2_

The transformations of alkoxy groups was studied in situ in IR cells at two different experimental conditions. In one series of experiments, alkoxy groups were generated by the adsorption of excess of ethanol (ca. 10 Tr in gas phase) followed by evacuation at 370 K (gas phase and weakly bonded ethanol molecules were removed). The cell with chemisorbed ethanol (mostly at the form of ethoxy groups) was subsequently heated to 410 and 470 K. After each step, the cell was cooled to room temperature and the IR spectrum was recorded. In the second experimental series the procedure was similar, the only difference was the lack of the desorption step. The oxides with chemisorbed ethanol were heated at the presence of gaseous ethanol (ca. 10 Tr).

**Figure 6:**
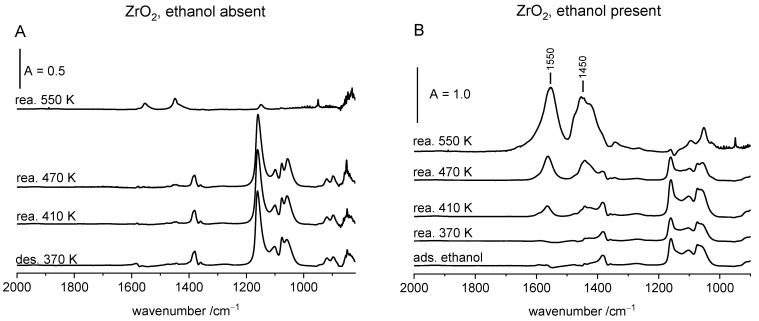

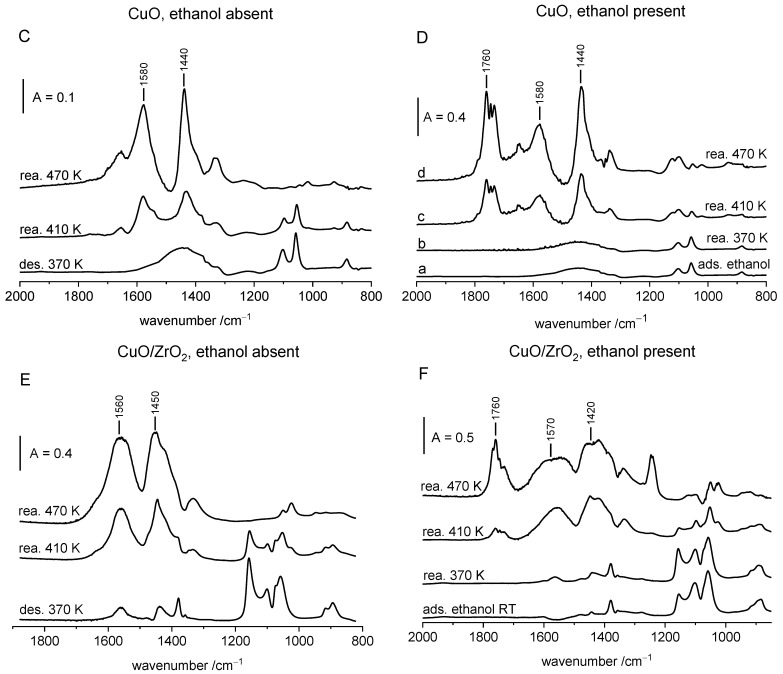
The spectra recorded upon the adsorption of ethanol on ZrO_2_, CuO and CuO/ZrO_2_ at room temperature and subsequent evacuation at 370 K (gaseous ethanol absent) (**A**,**C**,**E**) or upon the adsorption without evacuation (gaseous ethanol 15 Tr. present) (**B**,**D**,**F**). Spectra were recorded upon heating to 370, 410, 470 and (for ZrO_2_) 550 K and subsequent cooling to RT. The spectra of gaseous ethanol were subtracted.

The results concerning ZrO_2_ are presented in Figure 6A,B. In the absence of gaseous ethanol the spectrum of ethoxy groups practically does not change up to 470 K (Figure 6A), evidencing their high stability. Only above this temperature do they decompose, producing ethene and restoring all the hydroxyl groups (Figure 3). Small amount of acetate ions (IR bands 1450 and 1550 cm^−1^) were produced, evidencing that small amount of ethoxy groups was oxidized at 550 K. A somewhat different situation was observed if ethanol was present in the gas phase (Figure 6B). The acetate ions appeared already at 410 K and their amount increased at 470 K. Generally, the amount of acetate ions is significantly bigger than without gaseous ethanol.

The results obtained with CuO are presented in Figure 6C,D. The adsorption of ethanol causes the appearance of the bands at 884, 1058 and 1101 cm^−1^, typical of ethoxy groups of type II (bidental) The heating of these species to 410 and 470 K results in the formation of acetate ions (the bands 1440 and 1580 cm^−1^). In the experiment in which ethanol was absent in the cell, only acetate ions were formed. In the presence of ethanol in gas phase, acetaldehyde (bands around 1760 cm^−1^) was formed beside the acetate ions.

A similar situation was observed for CuO/ZrO_2_ (Figure 6E,F). In the absence of ethanol in the gas phase, only the acetate ions were formed, but in the presence of gaseous ethanol, both acetate ions and acetaldehyde were produced.

By summing up, it can be said that on in the case of all studied oxides, ethoxy groups were oxidized to acetate ions. Oxygen atoms originated from oxide surface. The following mechanism can be proposed: CH_3_CH_2_-O-M + 2O = CH_3_COO^-^ + M^+^ + H_2_O. On the other hand, acetaldehyde is formed directly from ethanol. Ethoxy groups cannot be transformed to acetaldehyde, because in the experiments in which gaseous ethanol was removed from the cell by evacuation and only ethoxy groups were present (Figure 6C,E), acetaldehyde was not produced (the bands around 1750 cm^−1^ were absent). According to several authors, acetaldehyde can be obtained from ethanol according to two mechanisms: by oxidation with the formation of water or by dehydrogenation with hydrogen production. According to our earlier studies [29] acetaldehyde on CuO is formed without the production of hydrogen, but with the formation of water evidencing an oxidation mechanism. On the other hand, on CuO/ZrO_2_, both hydrogen and water were produced, evidencing both routes of aldehyde formation [29]. Several authors discussed the mechanism of ethanol transformation to acetaldehyde. These authors [23,24] proposed that ethanol was first transformed into ethoxy groups which were next transformed to acetaldehyde with the release of hydrogen. The results obtained in our study support an alternative interpretation, according to which the ethoxy groups that formed on our oxides produced acetate ions. Acetaldehyde was formed only if molecular ethanol was present.

## 3. Materials and Methods

Pure ZrO_2_ was synthesized by dropwise addition of 30 wt% of ammonia (ACS reagent, Sigma-Aldrich, Poznan, Poland) to the 0.5 M solution of ZrO(NO_3_)_2_ · xH_2_O, (x = 5.7 calculated from DSC/TG analysis, 99%, Sigma-Aldrich) at pH = 1.5. The precipitated material was heated under reflux for 48 h. Next, the obtained product was filtered, washed with H_2_O, dried at 373 K and calcined at 887 K. CuO standard (ACS reagent, ≥99.0%) was purchased from Merck, Warsaw, Poland.

The CuO/ZrO_2_ catalyst was synthesized via co-precipitation method at pH = 7, using Na_2_CO_3_ (anhydrous, 99.5–100.5%, Sigma-Aldrich) as precipitating agent. The CuO to ZrO_2_ weight ratio was fixed to 2:3. Cations in the form of nitrates (Cu(NO_3_)_2_ · 3H_2_O, puriss. p.a., 99–104%, Sigma-Aldrich, ZrO_2_(NO_3_)_2_ · 5.7H_2_O) and Na_2_CO_3_ were simultaneously added dropwise into the beaker containing 100 mL of deionized water at 333 K. The mixture was vigorously stirred during the precipitation. Next, the precipitate was washed by five-time centrifugation at 4200 rpm, dried at 373 K and calcined at 823 K for 3 h.

The X-ray diffraction patterns were recorded according to the method described elsewhere [39]. The crystallite sizes of CuO and ZrO_2_ for CuO/ZrO_2_ and the crystallite sizes of monoclinic and tetragonal ZrO_2_ phases for pure ZrO_2_ were calculated according to the Sherrer equation. In the case of pure ZrO_2_, the volume fraction of monoclinic ZrO_2_ and tetragonal ZrO_2_ were calculated based on the XRD signal intensities characteristic for the monoclinic phase (−111) at 2θ = 28.2° and (111) at 2θ = 31.5° and for the tetragonal phase (101) at 2θ = 30.2° [40].

For IR studies, all oxides were pressed into thin wafers of ca. 100–200 mg. Prior to IR experiments, wafers were evacuated in situ in an IR cell at 470 K for 30 min. Ethanol was adsorbed at room temperature and subsequently heated to 370, 410, 470, and 550 K. After each heating step, the cell was cooled to room temperature and IR spectrum was recorded. The spectra were recorded with a NICOLET 6700 spectrometer (Thermo Scientific, Cambridge, MA, USA) with the spectral resolution of 1 cm^−1^.

## 4. Conclusions

Ethoxy groups on ZrO_2_ are formed by the reaction of ethanol with surface Zr-OH groups with the formation of water. Terminal Zr-OH (IR band 3775 cm^−1^) reacts with ethanol firstly forming ethoxy groups type I (IR bands at 920, 1077 and 1160 cm^−1^) before tribridged surface hydroxyls react with ethanol in the next order, forming ethoxy groups of type II (IR bands 898, 1055 and 1100 cm^−1^). Ethoxy groups on ZrO_2_ were relatively stable up to 470 K. Above this temperature they decomposed releasing ethene and restoring Zr-OH. The interaction of ethanol with CuO resulted in the formation of ethoxy groups of type II (bidendate) for which the bands at 884, 1058 and 1101 cm^−1^ are characteristic. The spectrum of the species formed by the reaction of ethanol on CuO/ZrO_2_ is the superposition of the spectra of ethoxy groups on ZrO_2_ and on CuO, evidencing that no new phase was formed in these mixed oxides. The further reactions of ethoxy groups on CuO and CuO/ZrO_2_ produce acetate ions (IR bands at ca. 1450, 1580 cm^−1^) by the addition of oxygen from the surface. Acetaldehyde is also produced on CuO and CuO/ZrO_2_, but it is formed directly from ethanol, without the intermediacy of ethoxy groups.

## Data Availability

Not applicable.

## Sample Availability

Samples of the compounds are not available from the authors.
